# Nutrition and lung cancer: a case control study in Iran

**DOI:** 10.1186/1471-2407-14-860

**Published:** 2014-11-21

**Authors:** Mostafa Hosseini, Parisa Adimi Naghan, Ali Moghadas Jafari, Mahmoud Yousefifard, Shervin Taslimi, Kian Khodadad, Forouzan Mohammadi, Makan Sadr, Mansour Rezaei, Esmaeil Mortaz, Mohammad Reza Masjedi

**Affiliations:** Department of Epidemiology and Biostatistics, Faculty of Public Health, Tehran University of Medical Sciences, Tehran, Iran; Lung Transplantation Research Center, National Research and Institute of Tuberculosis and Lung Diseases (NRITLD), Masih Daneshvari Hospital, Shahid Beheshti University of Medical Sciences, Shaheed Bahonar Ave, Darabad, Tehran, 1955841452 Iran; Department of Emergency Medicine, School of Medicine, Bushehr University of Medical Sciences, Bushehr, Iran; Department of Physiology, School of Medicine, Tehran University of Medical Sciences, Tehran, Iran; Decision of Neurosurgery, University of Toronto, Toronto, Ontario Canada; Chronic Respiratory Diseases Research Center, National Research and Institute of Tuberculosis and Lung Diseases (NRITLD), Masih Daneshvari Hospital, Shahid Beheshti University of Medical Sciences, Tehran, Iran; Mycobacteriology Research Center, National Research and Institute of Tuberculosis and Lung Diseases (NRITLD), Masih Daneshvari Hospital, Shahid Beheshti University of Medical Sciences, Tehran, Iran; Tracheal Diseases Research Center, National Research Institute of Tuberculosis and Lung Disease (NRITLD), Shahid Beheshti University of Medical Sciences, Tehran, Iran; Nursing and Respiratory Health Management Research Center, National Research and Institute of Tuberculosis and Lung Diseases (NRITLD), Masih Daneshvari Hospital, Shahid Beheshti University of Medical Sciences, Tehran, Iran; Division of Pharmacology, Utrecht Institute for Pharmaceutical Sciences, Faculty of Science, Utrecht University, Utrecht, The Netherlands

**Keywords:** Lung cancer, Nutrition, Cancer risk

## Abstract

**Background:**

Despite many prospective and retrospective studies about the association of dietary habit and lung cancer, the topic still remains controversial. So, this study aims to investigate the association of lung cancer with dietary factors.

**Method:**

In this study 242 lung cancer patients and their 484 matched controls on age, sex, and place of residence were enrolled between October 2002 to 2005. Trained physicians interviewed all participants with standardized questionnaires. The middle and upper third consumer groups were compared to the lower third according to the distribution in controls unless the linear trend was significant across exposure groups.

**Result:**

Conditional logistic regression was used to evaluate the association with lung cancer. In a multivariate analysis fruit (P_trend_ < 0.0001), vegetable (P = 0.001) and sunflower oil (P = 0.006) remained as protective factors and rice (P = 0.008), bread (P_trend_ = 0.04), liver (P = 0.004), butter (P_trend_ = 0.04), white cheese (P_trend_ < 0.0001), beef (P_trend_ = 0.005), vegetable ghee (P < 0.0001) and, animal ghee (P = 0.015) remained as risk factors of lung cancer. Generally, we found positive trend between consumption of beef (P = 0.002), bread (P < 0.0001), and dairy products (P < 0.0001) with lung cancer. In contrast, only fruits were inversely related to lung cancer (P < 0.0001).

**Conclusion:**

It seems that vegetables, fruits, and sunflower oil could be protective factors and bread, rice, beef, liver, dairy products, vegetable ghee, and animal ghee found to be possible risk factors for the development of lung cancer in Iran.

## Background

Lung cancer is the most common cancer worldwide. It is the leading cause of cancer related mortality, comprising 17% of all cancer deaths. As it does not have good prognosis its incidence and mortality rates are similar with approximately 1.5 million cases per year. Lung cancer is more common in men and in Northern hemisphere [1] with roughly more than 46.8 incident cases per 100,000 population in Europe and United States. In Iran it is the fifth most common cancer [2] with an incidence rate of 4.7-9.2 per 100,000 people [1]. Lung cancer comprises the second highest cost of cancer per patient worldwide, which is approximately 6,181 dollars per month [3, 4].

Smoking is regarded as the most important risk factor for lung cancer. Other risk factors such as exposure to air pollution, heavy metals, passive smoking, inorganic dusts, chemical compounds, exposures to radon and asbestos, radiation, and indoor emissions from burning fuels and also dietary factors have been determined [1].

Dietary patterns with different constituents are closely related to different type of cancers. In this regard, low intake of fruits and vegetable were found to be associated with increased risk of cancer [5]. Vegetable, fruit, beef, and butter consumption have been implicated in lung cancer development [6, 7].

Although with a low five-year survival rate of 16% [8] it is one of the most preventable cancers [1]. Indeed, its prevention can highly decrease the burden of this disease [4]. Smoking is considered to have a pivotal role in the prevention of lung cancer among other candidate risk factors. Physical activity, lower consumption of saturated fat and calorie-dense foods, and high intake of vegetables and fruit could be other means to prevent lung cancer [1, 6]. On the other hand, dietary habit remains a potential risk factor which can be modified in advance to development of lung cancer [9].

Despite a great number of prospective and retrospective studies about the association of dietary habit and lung cancer the topic still remains controversial and more studies are necessary to confirm or reject the prior surveys.

The aim of the present study is to examine association between lung cancer and dietary factors including raw/cooked vegetables, cereals, red/white meat, fish, dairy product, fruits and different oils.

## Methods

The study conducted in 5 differently located university hospitals in Tehran, Iran. More detailed information about this study is given by Hosseini et al. [2, 10]. Briefly, 242 histologically and cytologically confirmed lung cancer cases and their 484 matched controls for age (±3 years), sex and place of residence, consecutively were enrolled to the study during October 2002 to October 2005. The eligibility criteria for patients were pathologic confirmation, capability for undergoing a 1.5 hour interview and not any suspicious lung cancer metastasis from a different tumor. The first control group consisted of patients treated at the hospitals, excluding those with neoplasm and respiratory diseases. The second control group consisted of healthy people visiting other than the cases or other cancer patients.

The participation rate for cases and controls were 91.3% and 91.1%, respectively. The Ethical Review Board of the National Research Institute of Tuberculosis and Lung Disease (NRITLD) approved the present study. The study was designed and conducted in accordance with the Helsinki declaration and written informed consent were obtained from all study participants.

Trained physicians interviewed all cases and controls with structured questionnaire containing demographic characteristics, habitual history (lifelong history of tobacco use, including cigarette smoking, using hookah [water pipe], smoking, and congestion of recreational drugs and alcohol consumption), medical and family history of cancer, dietary habits, occupational history, several proposed environmental risk factors, residential history, and general medical history.

The nutrition questionnaire was developed through literature review and focus group discussion. It contained seven different groups of food including raw vegetable (fresh herbs, cucumber, lettuce, carrot, radish, onion, tomatoes, cauliflower, and green paper), cooked vegetable (mushroom, potato, onions, tomato, peas and beans, carrot, radish, turnip, beetroot, squash, garlic, celery, cauliflower, cabbage, pumpkin, and spinach), cereal (rice and bread), meat (sheep, beef, chicken, sheep and beef liver, fish, and shrimp), egg, dairy product (milk, butter, white cheese, yogurt), fruit (peach, apple, watermelon, nectarine, citrus fruit, and banana), and oil (mixed vegetable oils, sunflower oil, cornflower oil, vegetable ghee and animal ghee). Both vegetable and animal ghee are used for cooking in Iran and consists of saturated fats [11]. Subjects were asked about frequency of consumption of individual foods with a question containing 6 possible answers (more than 1 time a day, approximately one time a day, 2–4 times a week, less than 1 time a week, less than 1 time a month, never).

All the demographic characteristics of participants in the study were gathered into one table. After stratifying the cases and controls into approximate thirds for the distribution of various food types in control group, the middle and upper third consumers were compared to the lower third. For some kind of foods, like bread, chicken, fish, and shrimp the middle and upper tertiles were equal so we decided to categorize them into levels of never, less than median, and more than median of controls. This strategy was not possible for bread because of very low sample size in one the stratum so we decided to divide it into four groups. Conditional logistic regression used to evaluate the association of different foods with lung cancer development. Linear trend across the exposure groups was also evaluated. When trend was significant across the exposure groups only the p value of trend was reported and for reducing type one error only trend was used in conditional logistic regression instead of indicator variables (dummy). All the possible factors with P value of less than 0.1 entered into multivariate model to adjust for different food confounders. Two-sided P-values <0.05 were considered statistically significant to retain independent factors. All statistical analyses were carried out using STATA version 9.0 (StataCorp, College Station, TX, USA).

## Results

From five university hospitals 242 lung cancer cases enrolled to this study and matched to the 484 controls for age (±3 years), sex, and place of residence during a 3 years period (2002–2005). Demographic characteristics of the cases and controls are presented in Table 1. The range of age for the cases and controls were 17–87 and 17–86 with the means (±SD) of 55.9 ± 13.0 and 59.4 ± 12.8 years old, respectively. The majority of participants were male. Most of the cases and their controls were Persian. The other main ethnic groups were Azeri, Kord, and Lur. Other ethnic groups like Turkmen, Baluch, and Arabs were few so were combined together. Most of the participants were married. Education was categorized into two groups of less and more than 5 years of schooling. Overall, 66.5% of cases (85.4% of men and 14.1% of women) and 40.3% of controls (52.2% of men and 7.1% of women) were smokers.

**Table 1:** **Demographic characteristics of cases and matched controls, by numbers (%)**

Characteristics		Controls (%)	Cases (%)	OR (95% CI)	P_value
Age (Mean ± SD)					
	Male	60.8 ± 12.1 yr	61.3 ± 12.3 yr	-	-
	Female	55.4 ± 14.0 yr	55.9 ± 14.2 yr	-	-
	Total	59.4 ± 12.8 yr	55.9 ± 13.0 yr	-	-
Sex					
	Male	356 (73.6)	178 (73.6)	-	-
	Female	128 (26.4)	64 (26.4)	-	-
Ethnicity					
	Persian	256 (52.9)	109 (45.0)	Referent	-
	Azeri	129 (26.7)	83 (34.3)	1.8 (1.2-2.7)	0.007
	Kurd	21 (4.3)	10 (4.1)	1.1 (0.4-3.1)	0.85
	Lur	24 (5.0)	8 (3.3)	0.3 (0.1-1.5)	0.15
	Other	54 (11.1)	32 (13.3)	1.5 (0.8-2.8)	0.17
Marital status					
	Married	456 (94.2)	218 (90.1)	Referent	-
	Unmarried	28 (5.8)	24 (9.9)	2.0 (1.1-3.8)	0.03
Education					
	≥5-8 yr	314 (64.9)	116 (47.9)	Referent	-
	Nil & <5 yr	170 (35.1)	126 (52.1)	2.3 (1.6-3.2)	<0.0001
Smoking					
	Non-smoker	289 (59.7)	81 (33.5)	Referent	-
	Smoker	195 (40.3)	161 (66.5)	4.7 (3.0-7.2)	<0.0001

In the bivariate analyses we evaluated the possible cofounders (demographic and cigarette smoking) and found that being Azeri, unmarried, less educated (<5 years of schooling), and cigarette smoker were associated with the risk of lung cancer (P < 0.05). Therefore, the following analysis of the dietary habits was adjusted for the effect of these factors.

The results of the bivariate analyses showed nearly the same effect size (data is not shown) for different raw vegetables (fresh herbs, cucumber, lettuce, carrot, radish, onion, tomatoes, cauliflower, and green paper), cooked vegetables (mushroom, potato, onions, tomato, peas and beans, carrot, radish, turnip, beetroot, squash, garlic, celery, cauliflower, cabbage, pumpkin and spinach), and also different fruits (peach, apple, watermelon, nectarine, citrus fruit, and banana), to avoid type one error because of multiple comparisons we decided to merge all individual foods in each category so their consumption for each person were averaged for each raw and cooked vegetables and fruits.

As Table 2 shows, consumption of the moderate and high level of raw vegetables was significantly associated with lower risk of lung cancer (adjusted OR = 0.31; 95% CI: 0.20-0.48 and adjusted OR = 0.52; 95% CI: 0.32-0.86, respectively). Overall, only middle level consumption of cooked vegetables was significantly associated with risk of lung cancer (adjusted OR = 2.02; 95% CI: 1.28-3.2, P = 0.003). In addition, consumption of fruits was showed negative trend with the risk of lung cancer (P_trend_ < 0.0001). Middle level of rice consumption was associated with the risk of lung cancer (adjusted OR = 1.49; 95% CI: 1.01-2.2). Bread intake also showed positive trend with the risk of lung cancer development (P_trend_ < 0.0001).

**Table 2:** **Use of vegetables, fruits and cereals in cases and matched controls**

Diet			Control (%)	Case (%)	OR ^a^ (95% CI)	P_value
**Vegetables**	**Raw**	Lower third	128 (26.8)	113 (48.3)	Referent	-
		Middle third	218 (45.7)	68 (29.1)	0.31 (0.20-0.48)	<0.0001
		Upper third	131 (27.5)	53 (22.6)	0.52 (0.32-0.86)	0.01
	**Cooked**	Lower third	174 (38.5)	67 (29.5)	Referent	-
		Middle third	115 (25.4)	86 (37.9)	2.02 (1.28-3.20)	0.003
		Upper third	163 (36.1)	74 (32.6)	1.39 (0.86-2.26)	0.176
**Fruits**		Lower third	166 (34.5)	128 (54.0)	Referent	-
		Middle third	161 (33.5)	68 (28.7)	0.60 (0.40-0.89)	0.01
		Upper third	154 (32.0)	41 (17.3)	0.31 (0.18-0.52)	<0.0001
					Test for trend: P < 0.0001	
**Cereals**	**Rice**	Lower third	85 (38.6)	67 (38.0)	Referent	-
		Middle third	110 (46.0)	86 (38.8)	1.49 (1.01-2.20)	0.04
		Upper third	44 (18.4)	74 (23.2)	0.97 (0.60-1.57)	0.90
	**Bread**	<four a month	17 (3.5)	5 (2.1)	Referent	-
		2-4 a week	57 (11.8)	12 (5.0)	0.96 (0.28-3.27)	0.95
		Once a day	154 (31.9)	58 (24.4)	1.72 (0.57-5.23)	0.34
		>once a day	255 (52.8)	163 (68.5)	2.80 (0.94-8.31)	0.06
					Test for trend: P < 0.0001	

^**a**^Adjusted for smoking, ethnicity, marital status and education.

We evaluated the association of six different kind of meat consumption with the risk of lung cancer. Chicken was not associated with risk of lung cancer. Low consumption of fish and high consumption of beef were found as risk factors for lung cancer (P < 0.05). Liver and sheep were only found to be risk factors of lung cancer in middle level consumers (P < 0.0001). In contrast shrimp was protective factor in middle level exposure (adjusted OR = 0.42; 95% CI: 0.24-0.72). Only beef consumption had trend between different levels of consumption.

Egg consumption was not significant in any of levels. We evaluated four different type of dairy product (milk, butter, white, cheese, and yogurt). All dairy products were found to be risk factors for lung cancer and all of them had positive trend in three levels of exposure (Table 3).

**Table 3:** **Use of meat and dairy products in cases and matched controls**

Diet			Control (%)	Case (%)	OR ^a^ (95% CI)	P_value
**Meat**	**Sheep**	Lower third	190 (39.5)	69 (28.9)	Referent	-
		Middle third	212 (44.1)	130 (54.4)	21.7 (1.45-3.26)	<0.0001
		Upper third	79 (16.4)	40 (16.7)	1.54 (0.91-2.62)	0.11
	**Beef**	Lower third	265 (55.0)	105 (43.9)	Referent	-
		Middle third	183 (38.0)	104 (43.5)	1.41 (0.96-2.09)	0.08
		Upper third	34 (7.0)	30 (12.6)	2.67 (1.39-5.10)	0.003
					Test for trend: P = 0.002	
	**Chicken**	<four a month	156 (32.4)	74 (3.0)	Referent	-
		2-4 a week	278 (57.7)	141 (59.0)	1.19 (0.80-1.75)	0.40
		≥once a day	48 (9.9)	24 (10.0)	1.36 (0.73-2.53)	0.33
	**Sheep and beef liver**	Lower third	303 (63.0)	94 (39.5)	Referent	-
		Middle third	102 (21.2)	109 (45.8)	4.44 (2.82-7.01)	<0.0001
		Upper third	76 (15.8)	35 (14.7)	1.52 (0.88-2.62)	0.14
	**Fish**	Never	86 (17.9)	23 (9.6)	Referent	-
		<four a month	238 (49.5)	141 (59.0)	2.16 (1.24-3.77)	0.006
		≥four a month	157 (32.6)	75 (31.4)	1.71 (0.93-3.15)	0.08
	**Shrimps**	Never	328 (68.2)	189 (79.4)	Referent	-
		<four a month	98 (20.4)	25 (10.5)	0.42 (0.24-0.72)	0.002
		≥four a month	55 (11.4)	24 (10.1)	0.78 (0.40-1.53)	0.47
**Dairy products**	**Eggs**	Lower third	117 (49.2)	230 (47.8)	Referent	-
		Middle third	70 (33.2)	181 (37.6)	0.69 (0.46-1.03)	0.07
		Upper third	42 (17.6)	70 (14.6)	1.21 (0.72-2.04)	0.48
					Test for trend: P = 0.07	
	**Milk**	Lower third	157 (66.0)	346 (71.8)	Referent	-
		Middle third	61 (25.6)	94 (19.5)	1.97 (1.20-3.21)	0.007
		Upper third	20 (8.4)	42 (8.7)	2.64 (1.54-4.51)	<0.0001
					Test for trend : P < 0.0001	
	**Butter**	Lower third	172 (35.7)	49 (20.6)	Referent	-
		Middle third	194 (40.2)	99 (41.6)	1.70 (1.07-2.71)	0.025
		Upper third	116 24.1)	90 (37.8)	2.94 (1.79-4.82)	<0.0001
					Test for trend: P < 0.0001	
	**White cheese**	Lower third	49 (20.6)	261 (41.1)	Referent	-
		Middle third	143 (60.1)	185 (38.4)	3.87 (2.52-2.93)	<0.0001
		Upper third	46 (19.3)	36 (7.5)	6.52 (3.49-12.18)	<0.0001
					Test for trend: P < 0.0001	
	**Yogurt**	Lower third	151 (63.4)	337 (69.8)	Referent	-
		Middle third	67 (28.2)	108 (22.3)	2.37 (1.46-3.83)	<0.0001
		Upper third	20 (8.4)	38 (7.9)	2.98 (1.77-5.00)	<0.0001
					Test for trend: P < 0.0001	

^**a**^Adjusted for smoking, ethnicity, marital status and education.

We also evaluated association of five different oils consumption with lung cancer. Mixed vegetable oil (adjusted OR = 0.71), sunflower oil (adjusted OR = 0.13) and cornflower oil (adjusted OR = 0.11) found to be protective. In contrast, vegetable ghee (adjusted OR = 5.89) and animal ghee (adjusted OR = 1.89) found to be risk factors for development of lung cancer (Table 4).

**Table 4:** **Type of used oil used in case and controls**

Type of oil		Controls (%)	Case (%)	OR ^a^ (95% CI)	P_value
Mixed vegetable oils	No	290 (60.5)	167 (72.3)	Referent	-
	Yes	189 (39.5)	64 (27.7)	0.71 (0.48-1.06)	0.09
Sunflower oil	No	255 (47.2)	185 (82.3)	Referent	-
	Yes	252 (52.8)	40 (17.8)	0.13 (0.08-0.23)	<0.0001
Cornflower oil	No	272 (57.0)	201 (88.2)	Referent	-
	Yes	205 (43.0)	27 (11.8)	0.11 (0.06-0.21)	<0.0001
Vegetable ghee	No	15 (44.4)	31 (12.8)	Referent	-
	Yes	269 (55.6)	211 (87.2)	5.89 (3.6-9.62)	<0.0001
Animal ghee	No	308 (63.9)	110 (46.0)	Referent	-
	Yes	174 (36.1)	129 (54.0)	1.89 (1.30-2.74)	0.001

^**a**^Adjusted for smoking, ethnicity, marital status and education.

In a multivariate analysis we entered all associated factors with P value of less than 0.1 in the model. Fruit, beef, egg, milk, butter, white cheese, and yogurt had trend over the exposure levels so we entered them in the model considering their trend by assigning values of 1, 2 and 3 to their different level of use. As the levels of consumption of the other type of foods had not any trend we produced indicator variables when entering them in the model. This analysis also was adjusted for smoking, ethnicity, marital status and education.

Only 11 foods remained significant in the multivariate analysis. Fruit (OR_trend_ = 0.44) and sunflower oil (OR = 0.28) found to be protective and bread (OR_trend_ = 1.54), butter (OR_trend_ = 1.69), white cheese (OR_trend_ = 3.09), beef (OR_trend_ = 2.13), vegetable ghee (OR = 7.71), and animal ghee (OR = 2.27) found to be risk factor of lung cancer. Middle third level exposure to liver and rice with OR of 3.03 and 1.84 were risk factors and raw vegetable (OR = 0.42) was protective factor for lung cancer development. Detailed information on result of multivariate analysis is presented in Table 5.

**Table 5:** **Dietary risk factors for lung cancer; result of multivariate conditional logistic regression**

Diet		OR ^a^	SE	95% CI	P_value
**Raw vegetables**	Lower third	Referent	-	-	-
	Middle third	0.42	0.10	0.26-0.69	0.001
	Upper third	NS	-	-	-
**Fruit**		0.44	0.07	0.31-0.63	<0.0001
**Rice**	Lower third	Referent	-	-	-
	Middle third	1.84	0.42	1.17-2.89	0.008
	Upper third	NS	-	-	-
**Bread**		1.54	0.32	1.02-2.32	0.04
**Sheep and beef liver**	Lower third	Referent	-	-	-
	Middle third	3.03	1.16	1.44-6.40	0.004
	Upper third	NS	-	-	-
**Butter**		1.69	0.43	1.03-2.78	0.04
**White cheese**		3.09	0.91	1.73-5.50	<0.0001
**Beef**		2.13	0.57	1.25-3.61	0.005
**Sun flower oil**		0.28	0.13	0.11-0.70	0.006
**Vegetable ghee**		7.71	3.50	3.17-18.74	<0.0001
**Animal ghee**		2.27	0.77	1.17-4.40	0.015

^**a**^Adjusted for smoking, ethnicity, marital status and education.

## Discussion

In a multivariate analysis adjusted for ethnicity, marital status, and education and all possible related foods with lung cancer fruit, vegetable, and sunflower oil remained as a protective factor and rice, bread, liver, butter, white cheese, beef, vegetable ghee, and animal ghee remained as a risk factor of lung cancer. Generally, we found positive trend between consumption of egg, beef, bread, and dairy product (milk, butter, white cheese, and yogurt) and lung cancer. In contrast, fruits were the only food which were inversely related to lung cancer.

Raw vegetable and fruit found to be protective factors for development of lung cancer in our study. Data about association of vegetable and fruit consumption and lung cancer development is still controversial. A multi-center cohort study in Europe that investigated relation between different type of cancer and dietary habits, nutrition, and lifestyle on 1,939,011 subjects aged 25–70, concluded that only fruit intake was inversely related to the risk of lung cancer [12]. Another cohort study on 121,700 women (30–55 years ages) and 51,529 men (40–75 years ages) demonstrated that the fruit and vegetable intake reduced risk of lung cancer only in women [6]. Skuladottir et al. in 2004, in a prospective cohort study on 80,996 men and 79,729 women aged 50–64 years suggested that high intake of fruit, vegetables, and all plant food was associated with risk of lung cancer only in high consumers of plant foods and vegetables [13]. A systematic review investigated 25 case–control and 11 cohort studies on the effect of vegetables and fruits on lung cancer, all 25 case–control and 11cohort studies except 2 concluded that vegetable intake was a protective factor for development of lung cancer [14].

The mechanisms by which fruit and vegetable may act are not clearly understood yet [15]. However, it was suggested that vegetables and fruits consist of micronutrients that can interfere with detoxification enzymes and immune system, inhibit cell proliferation, induct cell differentiation, inhibit oncogene expression, induct cell-cycle arrest, induct apoptosis, and several other mechanisms. These compounds consist of antioxidant micronutrients such as carotenoids as well as other anticarcinogenic agents [16, 17]. In addition, flavonoids in vegetables, which scavenge free radicals, have been added to the list of protective compounds. Recent studies showed that phytoestrogens and Glucosinolates hydrolysis products are other potential micronutrients in vegetables and fruits that may be preventive against cancer [18, 19].

There is paucity in evaluating the cooked vegetable consequent effect on lung cancer. A review on two case control and one cohort studies concluded no relation between cooked vegetable and lung cancer as well [20]. Our study consistently showed that cooked vegetable consumption is not associated with the risk of lung cancer.

Between beef and sheep only beef remained significant in multivariate analysis. Inconsistent results obtained from different located studies about relation of meat and lung cancer. Cross et al. in their cohort study on approximately 500,000 people aged 50–71 have shown a positive association between red meat and risk of lung cancer [21]. Oppositely, a recent cohort study with 99,579 participants demonstrated no relation between type and cooking method of meat (red or processed) and lung cancer [22]. Heterocyclic amines (HCAs) and polycyclic aromatic hydrocarbons (PAHs), which are produced in high temperature cooking method of meat, have been established carcinogenic effect [23, 24]. Moreover, red meat as a source of saturated fat and iron have been found to be related with cancer induction [25].

Fish was not a risk factor for lung cancer in separate analysis with adjusting for ethnicity, marital status, and education analysis in our study. Contribution of fish in lung cancer is still the matter of debate. A review on effect of omega-3 in cancer reported the result of three cohort studies with three different protective, provocative and not relating effect of fish consumption as a proxy of omega 3 with development of lung cancer [26].

Brennan et al. in a multicenter case–control study in nonsmoker cases with accounting for several confounders demonstrated that liver and egg consumption are not related to lung cancer development [27]. Consistently, we did not find significant association between egg consumption with lung cancer however sheep and beef liver were found to be a risk factor for lung cancer in our study. Sheep and cows’ liver contains heavy metals and other poisons [28]. Liver is responsible for metabolizing and detoxifying food, antibiotics, vaccine ingredients, pesticide over-spray, tainted water, and the synthetic hormones that are frequently given to farm animals [29, 30]. This component may influence cancer induction.

It is not exactly obvious what the exact relation between lung cancer and dairy products is. In a separate analysis with adjusting for ethnicity, marital status and education all dairy product including milk, butter, white cheese and yogurt found to be a risk factor of lung cancer with positive trend in higher level consumers. Although only butter and white cheese remained in the multivariate model. A case control study enrolled 377 cases and 377 controls, found that after adjusting for body-mass index, family history of lung cancer, total energy intake and smoking, milk consumption were positively associated with lung cancer [31]. Furthermore, a multicenter study demonstrated that high intake of butter-increased risk of small cell carcinoma type of lung cancer [27]. Oppositely, a cohort study on 50,000 participant after 65-y follow-up, suggested that high intake of milk has an inverse relation to risk of lung cancer [9]. A multicenter case–control study in nonsmoker cases demonstrated that high consumption of cheese and margarine reduces risk of lung cancer [27]. The potential risk of dairy product for lung cancer may be due to the fact that they are the main source of calcium and a positive association between diets high in calcium and risk of cancer was established in a recent meta-analysis. In addition, presence of live microbes in dairy products and certain cheeses and their interaction with immune system, may influence induce of cancer [32].

According to literature, different oils have various effects on distinct cancers. Although several studies investigated the effect of oils in different cancers [33, 34] but there is paucity of studies on the effect of different oils in lung cancer. However, two animal models suggested a protective effect of olive oil and provocative effect of corn oil on lung cancer [35, 36]. We found that sunflower oil have a protective role but vegetable ghee and animal ghee a hazardous role on lung cancer. Sun flower oil contains low levels of gamma-tocopherol (an isoform of vitamin E) compared to other vegetable oils [37] but it is enriched by alpha-tocopherol. A recent study demonstrated that gamma-tocopherol can promote lung cancer [38] while alpha-tocopherol, found in olive and sunflower oils, decrease the risk of disease. It is associated with better lung function, and reduces inflammation [39]. In contrast, ghee especially vegetable ghee is full of Trans and saturated fat known to risk factors for cancer. However, Rani and Kansal demonstrated that cow ghee attenuates risk of breast cancer. The effect is mediated by decreased expression of cyclooxygenase-2 and increased expression of peroxisome proliferators activated receptor- γ (PPAR-γ) in mammary gland [40]. Further animal studies are needed to establish the protective or hazardous role of animal ghee on development of lung cancer.

Bread found to be a risk factor of lung cancer in multivariate analysis but rice was only associated with lung cancer in separate analysis with adjusting for ethnicity, marital status, and education. Although there are several studies about association of cereals with other cancers there is a scarcity in the evaluation of their relation with lung cancer. A cohort study in the US with a sample of 20,195 demonstrated that high consumption of cereal reduced risk of lung cancer [41]. Rice and bread consumed in Iran normally lack bran. Since most of antioxidant compounds are found in the bran cereal, removing it may eliminate preventive effect of these items against cancer.

It is so important to choose the control group in a case control study. We decided to match our control groups with our cases to reduce confounders and also choosing controls in a way to restrict selection bias in the study. As there is high controversy about the effect of different group of food with lung cancer we also analyzed some specific food to evaluate possible relation. Unfortunately, we did not gather extra information about method of cooking, fat concentration and accompanying foods, which are believed to be related to lung cancer.

As the review of literature shows, there is a high inconsistency in the effect of nutrition with lung cancer development. When looking precisely to different studies one realize that there are too many confounders that each study has to account them for. It has been proposed that susceptibility to environmental agents may be influenced by polymorphic metabolic genes in different ethnicities. On the other hand, nutrition is not usually a separate thing from activity, life style, and other exposures (for example air pollution, water contamination, and patient’s occupation) in the society which are believed to be involved in the development of lung cancer [42]. If we neglect this association there are also other things to be considered such as way of cooking, fat concentration and so on. Involvement of some kind of vitamins such as A, C, and E in addition to carrotenoinds and selenium have been proposed in the development of lung cancer [5, 43]. Different foods may contain different nutrients that may give rise to the dilution of results to a null hypothesis and make us erroneously think there is not any substantial relation or lead to controversies in the topic, but what may be true would be a nutrient we do not think about. Therefore, if we observe such inconsistencies in the literature, along with different strategies they used in their studies, misclassification of food and not accounting for possible confounders such as genetics may be the cause. Indeed, we strongly recommend restriction of studies to specific group of people to overcome these problems.

## Conclusions

We found that vegetables, fruits and sunflower oil could be protective factors against development of lung cancer. In contrast, bread, rice, beef, liver, dairy products, vegetable ghee, and animal ghee found to be possible risk factors of the development of lung cancer in Iran.

## References

[1] Garcia M, Jemal A, Ward EM, Center MM, Hao Y, Siegel RL, Thun MJ (2007). Global cancer facts & figures 2007. GA-Am Cancer Soci.

[2] Hosseini M, Naghan PA, Karimi S, SeyedAlinaghi SA, Bahadori M, Khodadad K, Mohammadi F, Keynama K, Masjedi MR (2009). Environmental risk factors for lung cancer in Iran: a case–control study. Int J Epidemiol.

[3] Chang S, Long SR, Kutikova L, Bowman L, Finley D, Crown WH, Bennett CL (2004). Estimating the cost of cancer: results on the basis of claims data analyses for cancer patients diagnosed with seven types of cancer during 1999 to 2000. J Clin Oncol.

[4] Kutikova L, Bowman L, Chang S, Long SR, Obasaju C, Crown WH (2005). The economic burden of lung cancer and the associated costs of treatment failure in the United States. Lung Cancer.

[5] Key TJ, Allen NE, Spencer EA, Travis RC (2002). The effect of diet on risk of cancer. Lancet.

[6] Feskanich D, Ziegler RG, Michaud DS, Giovannucci EL, Speizer FE, Willett WC, Colditz GA (2000). Prospective study of fruit and vegetable consumption and risk of lung cancer among men and women. JNCI.

[7] Takezaki T, Hirose K, Inoue M, Hamajima N, Yatabe Y, Mitsudomi T, Sugiura T, Kuroishi T, Tajima K (2001). Dietary factors and lung cancer risk in Japanese: with special reference to fish consumption and adenocarcinomas. BJC.

[8] Smith RA, Cokkinides V, von Eschenbach AC, Levin B, Cohen C, Runowicz CD, Sener S, Saslow D, Eyre HJ (2002). American cancer society guidelines for the early detection of cancer. CA-Cancer J Clin.

[9] van der Pols JC, Bain C, Gunnell D, Smith GD, Frobisher C, Martin RM (2007). Childhood dairy intake and adult cancer risk: 65-y follow-up of the Boyd Orr cohort. Am J Clin Nutr.

[10] Masjedi MR, Naghan PA, Taslimi S, Yousefifard M, Ebrahimi SM, Khosravi A, Karimi S, Hosseini M, Mortaz E (2012). Opium could be considered an independent risk factor for lung cancer: a case–control study. Respiration.

[11] Hu FB (2011). Globalization of diabetes the role of diet, lifestyle, and genes. Diabetes Care.

[12] Miller AB, Altenburg HP, Bueno-de-Mesquita B, Boshuizen HC, Agudo A, Berrino F, Gram IT, Janson L, Linseisen J, Overvad K (2003). Fruits and vegetables and lung cancer: findings from the European prospective investigation into cancer and nutrition. Int J Cancer.

[13] Skuladottir H, Tjoenneland A, Overvad K, Stripp C, Christensen J, Raaschou-Nielsen O, Olsen JH (2004). Does insufficient adjustment for smoking explain the preventive effects of fruit and vegetables on lung cancer?. Lung Cancer.

[14] Riboli E, Norat T (2003). Epidemiologic evidence of the protective effect of fruit and vegetables on cancer risk. Am J Clin Nutr.

[15] Skuladottir H, Tjoenneland A, Overvad K, Stripp C, Olsen JH (2006). Does high intake of fruit and vegetables improve lung cancer survival?. Lung Cancer.

[16] Tarrazo-Antelo AM, Ruano-Ravina A, Abal Arca J, Barros-Dios JM (2014). Fruit and vegetable consumption and lung cancer risk: a case–control study in Galicia, Spain. Nutr Cancer.

[17] Galeone C, Negri E, Pelucchi C, La Vecchia C, Bosetti C, Hu J (2007). Dietary intake of fruit and vegetable and lung cancer risk: a case–control study in Harbin, northeast China. Ann Oncol.

[18] Keck A-S, Finley JW (2004). Cruciferous vegetables: cancer protective mechanisms of glucosinolate hydrolysis products and selenium. Integr Cancer Ther.

[19] Brennan P, Hsu CC, Moullan N, Szeszenia-Dabrowska N, Lissowska J, Zaridze D, Rudnai P, Fabianova E, Mates D, Bencko V (2005). Effect of cruciferous vegetables on lung cancer in patients stratified by genetic status: a mendelian randomisation approach. Lancet.

[20] Link LB, Potter JD (2004). Raw versus cooked vegetables and cancer risk. Cancer Epidem Biomar.

[21] Cross AJ, Leitzmann MF, Gail MH, Hollenbeck AR, Schatzkin A, Sinha R (2007). A prospective study of red and processed meat intake in relation to cancer risk. Plos Med.

[22] Tasevska N, Cross AJ, Dodd KW, Ziegler RG, Caporaso NE, Sinha R (2011). No effect of meat, meat cooking preferences, meat mutagens or heme iron on lung cancer risk in the prostate, lung, colorectal, and ovarian cancer screening trial. Int J Cancer.

[23] Roth MJ, Wei WQ, Baer J, Abnet CC, Wang GQ, Sternberg LR, Warner AC, Johnson LL, Lu N, Giffen CA (2009). Aryl hydrocarbon receptor expression is associated with a family history of upper gastrointestinal tract cancer in a high-risk population exposed to aromatic hydrocarbons. Cancer Epidem Biomar.

[24] Sinha R, Kulldorff M, Curtin J, Brown CC, Alavanja MCR, Swanson CA (1998). Fried, well-done red meat and riskof lung cancer in women (United States). Cancer Causes Control.

[25] Tudek B, Swoboda M, Kowalczyk P, Olinski R (2006). Modulation of oxidative DNA damage repair by the diet, inflammation and neoplastic transformation. J Physiol Pharmacol.

[26] MacLean CH, Newberry SJ, Mojica WA, Khanna P, Issa AM, Suttorp MJ, Lim YW, Traina SB, Hilton L, Garland R (2006). Effects of omega-3 fatty acids on cancer risk: a systematic review. JAMA.

[27] Brennan P, Fortes C, Butler J, Agudo A, Benhamou S, Darby S, Gerken M, Jökel KH, Kreuzer M, Mallone S, Nyberg F, Pohlabeln H, Ferro G, Boffetta P (2000). A multicenter case–control study of diet and lung cancer among non-smokers. Cancer Causes Control.

[28] Akan J, Abdulrahman F, Sodipo O, Chiroma Y (2010). Distribution of heavy metals in the liver, kidney and meat of beef, mutton, caprine and chicken from Kasuwan Shanu market in Maiduguri Metropolis, Borno State, Nigeria. Res J Appl Sci Engineer Technol.

[29] Schneider PD (2004). Preoperative assessment of liver function. Surg Clin North Am.

[30] Prosser CL (1991). Comparative animal physiology, environmental and metabolic animal physiology.

[31] De Stefani E, Fontham ET, Chen V, Correa P, Deneo-Pellegrini H, Ronco A, Mendilaharsu M (1997). Fatty foods and the risk of lung cancer: a case–control study from Uruguay. Int J Cancer.

[32] Lampe JW (2011). Dairy products and cancer. J Am Coll Nutr.

[33] Edem DO (2002). Palm oil: biochemical, physiological, nutritional, hematological, and toxicological aspects: a review. Plant Foods Hum Nutr.

[34] Willett WC (1995). Diet, nutrition, and avoidable cancer. Environ Health Pers.

[35] Parodi PW (1997). Cows’ milk fat components as potential anticarcinogenic agents. J Nutr.

[36] Smith TJ, Yang GY, Seril DN, Liao J, Kim S (1998). Inhibition of 4-(methylnitrosamino)-1-(3-pyridyl)-1-butanone-induced lung tumorigenesis by dietary olive oil and squalene. Carcinogenesis.

[37] Cook-Mills JM, Abdala-Valencia H, Hartert T (2013). Two faces of vitamin E in the lung. Am J Respir Crit Care Med.

[38] Sayin VI, Ibrahim MX, Larsson E, Nilsson JA, Lindahl P, Bergo MO (2014). Antioxidants accelerate lung cancer progression in mice. Sci Transl Med.

[39] Berdnikovs S, Abdala-Valencia H, McCary C, Somand M, Cole R, Garcia A, Bryce P, Cook-Mills JM (2009). Isoforms of vitamin E have opposing immunoregulatory functions during inflammation by regulating leukocyte recruitment. J Immunol.

[40] Rani R, Kansal VK (2011). Study on cow ghee versus soybean oil on 7, 12-dimethylbenz (a)-anthracene induced mammary carcinogenesis & expression of cyclooxygenase-2 & peroxisome proliferators activated receptor-γ in rats. Indian J Med Res.

[41] Breslow RA, Graubard BI, Sinha R, Subar AF (2000). Diet and lung cancer mortality: a 1987 National Health Interview Survey cohort study. Cancer Causes Control.

[42] Biesalski HK, Bueno MB, Chesson A, Chytil F, Grimble R, Hermus RJ, Kِhrle J, Lotan R, Norpoth K, Pastorino U (1998). European consensus statement on lung cancer: risk factors and prevention. lung cancer panel. CA-Cancer J Clin.

[43] Yong LC, Brown CC, Schatzkin A, Dresser CM, Slesinski MJ, Cox CS, Taylor PR (1997). Intake of vitamins E, C, and A and risk of lung cancer the NHANES I epidemiologic followup study. Am J Epidemiol.

[CR44] The pre-publication history for this paper can be accessed here: http://www.biomedcentral.com/1471-2407/14/860/prepub

